# The impact of the COVID-19 pandemic on people living with HIV: a cross-sectional study in Caracas, Venezuela

**DOI:** 10.1186/s12879-023-08967-6

**Published:** 2024-01-15

**Authors:** David A. Forero-Peña, Fhabián S. Carrión-Nessi, José L. Forero-Peña, Natasha A. Camejo-Ávila, Daniela L. Mendoza-Millán, Óscar D. Omaña-Ávila, Andrea L. Maricuto, Viledy L. Velásquez, Mario D. Mejía-Bernard, Carlis M. Rodriguez-Saavedra, María V. Marcano-Rojas, Yoesmir Contreras, Luis J. Guerra, María F. Alvarado, Martín Carballo, Jocays Caldera, Rafael N. Guevara, María C. Redondo, María E. Landaeta

**Affiliations:** 1 Biomedical Research and Therapeutic Vaccines Institute, Ciudad Bolivar, Venezuela; 2 Infectious Diseases Department, University Hospital of Caracas, Caracas, Venezuela; 3 “Luis Razetti” School of Medicine, Central University of Venezuela, Caracas, Venezuela

**Keywords:** Human immunodeficiency virus, HIV, COVID-19, SARS-CoV-2, Clinical profile, Epidemiology, Venezuela

## Abstract

**Background:**

The coronavirus disease 2019 (COVID-19) pandemic has disrupted multiple health services, including human immunodeficiency virus (HIV) testing, care, and treatment services, jeopardizing the achievement of the Joint United Nations Programme on HIV/AIDS 90-90-90 global target. While there are limited studies assessing the impact of the COVID-19 pandemic on people living with HIV (PLHIV) in Latin America, there are none, to our knowledge, in Venezuela. This study aims to assess the impact of the COVID-19 pandemic among PLHIV seen at the outpatient clinic of a reference hospital in Venezuela.

**Methods:**

We conducted a cross-sectional study among PLHIV aged 18 years and over seen at the Infectious Diseases Department of the University Hospital of Caracas, Venezuela between March 2021 and February 2022.

**Results:**

A total of 238 PLHIV were included in the study. The median age was 43 (IQR 31–55) years, and the majority were male (68.9%). Most patients (88.2%, *n* = 210) came for routine check-ups, while 28 (11.3%) were newly diagnosed. The majority of patients (96.1%) were on antiretroviral therapy (ART), but only 67.8% had a viral load test, with almost all (95.6%) being undetectable. Among those who attended regular appointments, 11.9% reported missing at least one medical consultation, and 3.3% reported an interruption in their ART refill. More than half of the patients (55.5%) had received at least one dose of the COVID-19 vaccine, while the rest expressed hesitancy to get vaccinated. Most patients with COVID-19 vaccine hesitancy were male (65.1%), younger than 44 years (57.5%), employed (47.2%), and had been diagnosed with HIV for less than one year (33%). However, no statistically significant differences were found between vaccinated patients and those with COVID-19 vaccine hesitancy. Older age was a risk factor for missing consultations, while not having an alcoholic habit was identified as a protective factor against missing consultations.

**Conclusion:**

This study found that the COVID-19 pandemic had a limited impact on adherence to medical consultations and interruptions in ART among PLHIV seen at the University Hospital of Caracas, Venezuela.

## Background

Human immunodeficiency virus (HIV) remains a significant global public health problem, with approximately 39 million people living with HIV (PLHIV) and 630,000 acquired immunodeficiency syndrome (AIDS)-related deaths in 2022 [1]. The introduction of antiretroviral therapy (ART) has been a game-changer in the fight against HIV. Highly effective drugs with improved pharmacokinetics and tolerability have enhanced the prognosis and quality of life for PLHIV, contributing to a 68.5% decline in AIDS-related deaths worldwide between 2004 and 2022 [1]. Since 2018, the World Health Organization (WHO) has recommended dolutegravir-based regimens as first- and second-line treatments for all PLHIV due to their high efficacy and favorable toxicity profile [3]. However, the use of these regimens has been primarily limited to high-income countries [4].

Venezuela has experienced the highest rate of ART interruptions among Latin American countries since 2016, with the situation worsening in 2017 and 2018 due to limited access to ART, with only 16% of patients receiving treatment by April 2018 [5]. However, through the efforts of nongovernmental organizations and the implementation of the Master Plan for the Strengthening of the Response to HIV, Tuberculosis, and Malaria in Venezuela [6], nationwide access to dolutegravir-based ART was resumed in February 2019. Despite this progress, monitoring of the efficacy and tolerability of this regimen in both treatment-experienced and newly diagnosed patients has been limited. Strict adherence to ART is crucial for maintaining an undetectable viral load, reducing the risk of progression to AIDS and transmission of HIV to sexual partners [7]. However, epidemiological surveillance related to access to diagnosis, treatment, and viral suppression remains limited in Venezuela.

The coronavirus disease 2019 (COVID-19) pandemic has had significant direct and indirect impacts on global health, particularly in low- and middle-income countries [8, 9], including Venezuela, where the effects are compounded by a complex humanitarian crisis, weakened health systems, and concurrent epidemics such as HIV, malaria, and tuberculosis [5]. In some countries, the COVID-19 pandemic has disrupted HIV testing, care [9–11], and treatment [12] services, potentially leading to increased HIV-related deaths and transmission, and jeopardizing progress towards the Joint United Nations Programme on HIV/AIDS 90-90-90 global target [13]. Moreover, despite that several studies showing that COVID-19 vaccination is effective in preventing these adverse outcomes [14–17], vaccine hesitancy remains a global problem [18], particularly among higher-risk populations such as PLHIV. Concerns about vaccine safety have been identified as a primary factor contributing to COVID-19 vaccine hesitancy among this population [19, 20].

Currently, there is limited information available on the impact of the COVID-19 pandemic on PLHIV in Venezuela. Additionally, the rate of COVID-19 vaccine hesitancy among this population is unknown. This study aims to assess the impact of the COVID-19 pandemic and COVID-19 vaccine hesitancy among PLHIV seen at the outpatient clinic of the Infectious Diseases Department at the University Hospital of Caracas during the period of the COVID-19 pandemic in Venezuela.

## Methods

### Study design and patients

A cross-sectional study was conducted between March 2021 and February 2022 at the outpatient clinic of the Infectious Diseases Department at the University Hospital of Caracas, Venezuela. This specialized outpatient clinic, which began operating in 1990, provides care for patients with HIV infection and is considered the largest in the country, having attended a total of 6,350 patients in 2022. The study included consecutive patients aged 18 years and over with either known HIV infection or newly diagnosed with a positive HIV result. According to the Statistics Department of the University Hospital of Caracas, in 2021 there were 5,346 outpatient consultations of PLHIV. To analyze this population with a 95% confidence interval and a margin of error of 5%, a sample size of at least 359 participants was required. The sampling method employed was non-probabilistic.

### Survey design and data collection

A data collection form was designed to gather both sociodemographic and clinical data. Sociodemographic data collected included age, sex, origin by state, education level, marital status, occupation, income in US$ per month, sexual orientation, and whether the individual had a stable partner. Clinical data collected included time since HIV diagnosis, comorbidities, sexually transmitted diseases (STDs) history, AIDS-associated diseases history, recommended vaccination history, current ART, previous ART regimen, post-ART viral load, post-ART CD4^+^ count, and weekly treatment adherence. Additionally, information related to COVID-19 and the COVID-19 pandemic was also collected. This included COVID-19 history and severity, COVID-19 vaccination status and reasons for COVID-19 vaccine hesitancy, problems related to compliance with consultations in the last 12 months, and ART refilling during the COVID-19 pandemic.

### Data analysis

Participant data were summarized using descriptive statistics, including mean, standard deviation (SD), median, interquartile range (IQR), and/or frequency, percentage (%). The normality of numeric variables was assessed using the Kolmogorov-Smirnov test. Univariable analyses were performed using tests such as the Mann-Whitney U test for numerical variables with a non-normal distribution, Student’s t-test for those with a normal distribution, and Pearson’s chi-squared and Fisher’s exact tests for categorical variables. *P* values less than 0.05 were considered significant. Statistically significant variables identified in the univariable analyses were included in a binomial logistic regression using the enter method to identify factors associated with missed consultations. The best model was selected based on the highest percentage of participants including goodness of fit, R^2^ Nagelkerke, and the Hosmer-Lemeshow test. Statistical analyses were performed using the Statistical Package for the Social Sciences version 26 (International Business Machines Corporation, Armonk, NY, USA).

## Results

### Sociodemographic and epidemiologic characteristics of PLHIV

A total of 238 patients were analyzed, of which 210 came for a follow-up appointment, including eight who were returning to their controls after having abandoned treatment. The remaining 28 patients were coming to their first appointment for ART initiation. The median age of the patients was 43 (IQR 31–55) years, with the majority being male (68.9%) and heterosexual (50%). Most patients came from the Capital District (45.8%) and Miranda state (45.4%). Almost half of them had a steady partnership, and among these partners, 52.1% (*n* = 60/115) had negative HIV serology, 40.9% (*n* = 47/115) had positive HIV serology, and 7% (*n* = 8/115) were unaware of their HIV status. Additional sociodemographic data may be found in Table 1.

**Table 1:** Sociodemographic characteristics of patients with HIV infection

	*n* = 238
*Age, median (IQR), years*	43 (31–55)
*Sex, male/female (%)*	164/74 (68.9/31.1)
*Origin by state, n (%)*	
Miranda	110 (46.2)
Capital District	107 (44.9)
La Guaira	8 (3.4)
Carabobo	5 (2.1)
Aragua	3 (1.3)
Other state	5 (2.1)
*Education level, n (%)*	
None	3 (1.3)
Primary school	68 (28.6)
High school	69 (29)
University	98 (41.1)
*Marital status, n (%)*	
Single	153 (64.3)
Common-law marriage	47 (19.8)
Married	21 (8.8)
Widower	11 (4.6)
Divorced	6 (2.5)
*Occupation, n (%)*	
Employee	103 (43.3)
Independent	80 (33.6)
Unemployed/Retired	55 (23.1)
*Income in US$ per month, n (%)*	
≤$20	72 (30.2)
$21–100	107 (45)
$101–200	35 (14.7)
≥$201	24 (10.1)
*Sexual orientation, n (%)*	
Heterosexual	119 (50)
Homosexual	95 (39.9)
Bisexual	24 (10.1)
*Stable partner, yes/no (%)*	115/123 (48.3/51.7)

IQR: interquartile range

### Clinical information and ART history

The most frequent comorbidity among patients was hypertension (11.8%, *n* = 28), followed by osteoporosis (5%, *n* = 12), asthma (3.4%, *n* = 8), and diabetes (2.5%, *n* = 6). In relation to STDs history, syphilis was identified as the most frequent (19.3%, *n* = 46), followed by human papillomavirus infection. A tuberculosis history, both intra- and extrapulmonary, was the most frequent AIDS-associated disease (10.5%, *n* = 25). In relation to compliance with the recommended vaccines for PLHIV, the least compliant was the pneumococcal vaccine (Table 2).

**Table 2:** Clinical characteristics of patients with HIV infection

	*n* = 238
*Time since HIV diagnosis, n (%)*	
≤ 1 year	60 (25.2)
2–5 years	41 (17.2)
6–10 years	37 (15.6)
≥ 11	100 (42)
*Comorbidities, yes (%)*	
Hypertension	28 (11.8)
Osteoporosis	12 (5.0)
Asthma	8 (3.4)
Diabetes	6 (2.5)
Dyslipidemia	7 (2.9)
Cancer	5 (2.1)
*STDs history, yes (%)*	
Syphilis	46 (19.3)
Genital HPV	34 (14.3)
Gonorrhea	6 (2.5)
Hepatitis B	6 (2.5)
Hepatitis C	3 (1.3)
Genital herpes	3 (1.3)
*AIDS-associated diseases history, yes (%)*	
Pulmonary TB	14 (5.9)
Toxoplasmic encephalitis	10 (4.2)
Kaposi’s sarcoma	7 (2.9)
Histoplasmosis	8 (3.4)
Cryptococcosis	4 (1.7)
Ocular cytomegalovirus	4 (1.7)
Nodal TB	3 (1.3)
*Recommended vaccination history*	*n* = 210
Hepatitis B vaccine, yes (%)	122 (58.1)
Influenza vaccine, yes (%)	101 (48.1)
Pneumococcal vaccine, yes (%)	73 (34.8)

HIV: human immunodeficiency virus; STDs: sexually transmitted diseases; HPV: human papillomavirus; AIDS: acquired immunodeficiency syndrome; TB: tuberculosis

Regarding ART, excluding newly diagnosed patients who were started on treatment for the first time (*n* = 28), almost all patients (96.1%, *n* = 202) were on ART. The majority of these patients (91%) were receiving the combination of tenofovir disoproxil fumarate, lamivudine, and dolutegravir (TLD), followed by the combination of abacavir, lamivudine, and dolutegravir (8.9%). Only eight patients with a known HIV diagnosis were off treatment because they had discontinued it. One hundred and fifty-five patients had been previously exposed to ART, with a mean of 1.5 (SD 1.5, range: 1–7) previous regimens. The most frequent previous regimen was the combination of two nucleoside reverse transcriptase inhibitors (NRTIs) plus non-nucleoside reverse transcriptase inhibitors (NNRTIs) in 51% of patients (*n* = 79). Out of the patients on ART (*n* = 202), only 137 had available viral load data (67.8%) and almost all of these patients had undetectable viral loads (< 200 copies of RNA, 95.6%, *n* = 131). The undetectability rate was 95.8% (*n* = 115/120) for patients on the TLD regimen and 94.1% (*n* = 16/17) for those on the abacavir, lamivudine, and dolutegravir regimen. Only 22 out of 202 patients (10.9%) had a CD4^+^ count available. Finally, adequate adherence was observed in most patients (84.1%, *n* = 170/202), but 32 patients (15.8%) reported skipping at least one dose per week (Table 3).

**Table 3:** Characteristics related to ART

Current ART, n (%)	*n* = 202
2 NRTI + INSTI (TLD)	184 (91)
2 NRTI without TDF + INSTI	18 (8.9)
*Previous ART regimen, n (%)*	*n* = 155
2 NRTIs + INSTI	14 (9.0)
2 NRTIs + PI	60 (38.7)
2 NRTIs + NNRTI	78 (50.3)
Other	3(1.9)
*Post-ART viral load, n (%)*	*n* = 202
≤ 20	98 (48.5)
21–199	33 (16.3)
200–999	4 (2.0)
≥ 1000	2 (1.0)
Not available	65 (32.2)
*CD4*^+^ *post-ART, n (%)*	*n* = 202
≤ 200	6 (2.9)
201–350	3 (1.5)
351–500	4 (2.0)
≥ 501	9 (4.5)
Not available	180 (89.1)
*Weekly treatment adherence (tablet ingested/prescribed)*	*n* = 202
4/7	1 (0.5)
5/7	4 (2.0)
6/7	27 (13.4)
7/7	170 (84.1)

NRTIs: nucleoside reverse transcriptase inhibitors; NNRTI: non-nucleoside reverse transcriptase inhibitor; PI: protease inhibitor; INSTI: integrase strand transfer inhibitors; TLD: dolutegravir + lamivudine + tenofovir disoproxil fumarate; TDF: tenofovir disoproxil fumarate; ART: antiretroviral treatment

### COVID-19 vaccine hesitancy and the impact of the COVID-19 pandemic on PLHIV

Only 43 patients (18.1%) reported having had severe acute respiratory syndrome coronavirus 2 (SARS-CoV-2) infection, most of them diagnosed clinically (60.5%) without a confirmatory test and experiencing mild clinical manifestations (83.7%). Regarding COVID-19 vaccination, more than half of the patients (55.5%, *n* = 132) had received at least one dose of the vaccine (Table 4). Of these patients, 25 (18.9%) had received one dose, 92 (69.7%) had received two doses, and only 15 (11.4%) had received three doses. Among the unvaccinated group (44.5%, *n* = 106), all were hesitant to get vaccinated. Of these patients, 77 (72.6%) were unsure about getting vaccinated and wanted to consult with their doctor, while 29 (27.4%) preferred not to be vaccinated for distinct reasons: 23 (79.3%) expressed fear, four (13.8%) reported distrust, and four (13.8%) stated that they did not need it.

**Table 4:** Characteristics associated with SARS-CoV-2 infection and the COVID-19 pandemic

	*n* = 238
*SARS-CoV-2 infection history, yes (%)*	43 (18.1)
Diagnosis by RT-PCR	9 (20.9)
Diagnosis by antigen	2 (4.7)
Diagnosis by antibodies	6 (13.9)
Diagnosis by clinical	26 (60.5)
*COVID-19 severity, n (%)*	*n* = 43
Asymptomatic	2 (4.7)
Mild	36 (83.7)
Moderate	2 (4.7)
Severe	3 (6.9)
*COVID-19 vaccination, yes (%)*	132 (55.5)
BBIBP-CorV/Sinopharm	102 (77.3)
Gam-COVID-Vac/Sputnik-V	26 (19.7)
CIGB-66/Abdala (vaccine candidate)	2 (1.5)
ChAdOx1-S/nCoV-19/AstraZeneca	1 (0.75)
BNT162b2/Pfizer-BioNTech	1 (0.75)
*Problems during the COVID-19 pandemic*	*n* = 210
With HIV consultations, yes (%)	25 (11.9)
With ART supply, yes (%)	7 (3.33)
*Consultations in the last 12 months, n (%)*	*n* = 210
None	33 (15.7)
One visit	98 (46.7)
Two visits	69 (32.9)
Three or more visits	10 (4.7)

SARS-CoV-2: severe acute respiratory syndrome coronavirus 2; COVID-19: coronavirus disease 2019; RT-PCR: reverse transcriptase polymerase chain reaction; HIV: human immunodeficiency virus; ART: antiretroviral treatment

Most patients with COVID-19 vaccine hesitancy were male (65.1%), younger than 44 years old (57.5%), employed (47.2%), with a monthly income between US$21–100 (47.2%), and had been diagnosed with HIV for less than one year (33%), but there were no statistically significant differences when compared to the vaccinated group. The proportion of patients with an HIV diagnosis of less than one year was higher among those with vaccine hesitancy (33%) compared to vaccinated patients (18.9%). In contrast, the proportion of patients with a diagnosis of 16 or more years was more frequent among vaccinated patients (31.1%) compared to those with vaccine hesitancy (17%). However, these differences were not statistically significant (*p* = 0.053). Although the proportion of patients with comorbidities was higher in the vaccinated group (40.2%) compared to the vaccine hesitancy group (29.2%), there were no statistically significant differences between the two groups (*p* = 0.08) (Table 5).

**Table 5:** General characteristics of the COVID-19 vaccinated group and the COVID-19 vaccine hesitant group

Characteristics	Vaccinated group (*n* = 132, 55.5%)	Vaccine hesitant group (*n* = 106, 44.5%)	*p* value
*Sex, male/female (%)*	95/37 (72/28)	69/37 (65.1/34.9)	0.255^*^
*Age group, n (%)*			0.164^*^
< 43 years	64 (48.5)	61 (57.5)	
≥ 44 years	68 (51.5)	45 (42.5)	
*Occupation, n (%)*			0.676^†^
Employee	69 (52.3)	50 (47.2)	
Independent	35 (26.5)	29 (27.4)	
Unemployed/Retired	28 (21.2)	27 (25.5)	
*Income in US$ per month, n (%)*			0.229^*^
≤$20	38 (28.8)	34 (32.1)	
$21–100	57 (43.2)	50 (47.2)	
$101–200	25 (18.9)	10 (9.4)	
≥$201	12 (9.1)	12 (11.3)	
*Smoking habits, yes (%)*	47 (35.6)	28 (26.4)	0.129^*^
*Drinking habits, yes (%)*	5 (3.8)	6 (5.7)	0.546^†^
*Illicit drug use, yes (%)*	13 (9.8)	5 (4.7)	0.137^*^
*STDs history, yes (%)*	45 (34.1)	30 (28.3)	0.385^*^
*Time since HIV diagnosis, n (%)*			0.053^*^
≤ 1 year	25 (18.9)	35 (33)	
2–5 years	23 (17.4)	18 (17)	
6–10 years	20 (15.2)	17 (16)	
11–15 years	23 (17.4)	18 (17)	
≥ 16	41 (31.1)	18 (17)	
*Presence of at least one comorbidity, yes (%)*	53 (40.2)	31 (29.9)	0.167^*^
*AIDS-associated diseases history, yes (%)*	36 (27.3)	41 (38.7)	0.08^*^
*COVID-19 history, yes (%)*	24 (18.2)	19 (17.9)	0.992^*^

^*^Pearson’s chi-square test; ^†^Fisher’s exact test. STDs: sexually transmitted diseases; HIV: human immunodeficiency virus; AIDS: acquired immunodeficiency syndrome; COVID-19: coronavirus disease 2019

C

In relation to the impact of the COVID-19 pandemic on PLHIV who came to the consultation, only patients who came for control appointments were analyzed (*n* = 210). Of these patients, 11.9% (*n* = 25/210) reported having missed at least one medical consultation due to COVID-19 pandemic restrictions; of these patients, 92% (*n* = 23/25) missed their appointment due to restricted mobility, another 8% (*n* = 2/25) due to fear of becoming infected, and one because they were outside the country. The median number of consultations in the last 12 months for these patients was 1 (IQR 1–2) consultation per patient. A model was performed to evaluate factors associated with missed consultations (*p* = 0.01, R^2^ Nagelkerke = 0.34, Hosmer–Lemeshow test = 0.457), and found that older age was a risk factor for missing consultations (OR = 1.058, 95% CI = 1.009–1.11, *p* = 0.019). Additionally, not having an alcoholic habit was identified as a protective factor against missing consultations (OR = 0.012, 95% CI = 0.001–0.108, *p* < 0.001) (Table 6). Only 3.3% of patients reported interruption of ART refill due to the COVID-19 pandemic, all reporting that they were unable to claim their medication refill due to COVID-19 pandemic restrictions.

**Table 6:** Factors associated with missed visits during the COVID-19 pandemic

^1^	β	*p* value	OR (95% confidence interval)
*Age*	0.057	0.019	1.058 (1.009–1.11)
*Sex, male*	-1.126	0.074	0.324 (0.094–1.116)
*Occupation*			
Employee	0.684	0.368	1.982 (0.447–8.793)
Unemployed/Retired	0.597	0.472	1.816 (0.357–9.253)
*Origin by state*			
Capital District	0.025	0.98	1.025 (0.143–7.357)
Miranda	0.453	0.645	1.573 (0.229–10.801)
*Marital status*			
Single	0.851	0.496	2.342 (0.202–27.111)
Common-law marriage	1.145	0.403	3.141 (0.215–45.805)
Married	0.043	0.979	1.044 (0.04–27.087)
Divorced	0.76	0.65	2.139 (0.08–57.266)
*Smoking habits, no*	-0.347	0.567	0.707 (0.216–2.318)
*Alcoholic habits, no*	-4.384	< 0.001	0.012 (0.001–0.108)
*Illicit drug use, no*	-0.471	0.676	0.624 (0.069–5.676)
*Presence of at least one comorbidity, no*	0.745	0.255	2.107 (0.584–7.603)
*AIDS-associated diseases history, no*	0.903	0.161	2.468 (0.698–8.723)
*Weekly treatment adherence (tablet ingested/prescribed)*			
4/7	-20.104	1	0 (0)
5/7	1.3	0.389	3.668 (0.19–70.66)
6/7	-1.042	0.273	0.353 (0.055–2.27)

## Discussion

This study describes the epidemiological and clinical behavior of PLHIV at the Hospital University of Caracas, Venezuela and estimates the impact of the COVID-19 pandemic on disruptions in care, ART, and COVID-19 vaccine hesitancy. The majority of patients were young, employed men, consistent with previous reports [21–23]. Nearly half had a tertiary level of education, yet three-quarters earned less than US$100 per month, insufficient for access to basic food necessities [24]. Most patients were in heterosexual relationships [21, 22], with almost half reporting a stable partner. Nearly half of these partnerships were HIV serodiscordant, similar to other studies [25, 26]. Some studies reported substantial interruptions in pre-exposure prophylaxis (27.8–56%) during COVID-19 restrictions [27–30]. However, Venezuela does not have a pre-exposure prophylaxis program.

The impact of the COVID-19 pandemic on the diagnosis, care, and treatment of HIV infection has been extensively explored in other countries [31–34]. In general, new HIV diagnoses decreased by 12–45% [35–41]. In this study, a quarter of patients were recently diagnosed (< 1 year), emphasizing the importance of maintaining diagnostic and care activities during the COVID-19 pandemic. Adherence to ART and undetectability rates were similar to those reported in other Latin American countries such as Peru [42], Brazil [30, 43], Argentina [44], and globally during the COVID-19 pandemic [41, 43, 45]. Despite WHO recommendations for continuity of HIV services during the COVID-19 pandemic [46], care and treatment have faced challenges worldwide. Unlike other countries [41, 47–49], we did not use telemedicine due to barriers such as lack of equipment and inconsistent internet access. Instead, we maintained face-to-face consultations with strict biosecurity measures and provided ART refills for longer periods, as documented in other countries [50].

The COVID-19 pandemic has variably impacted clinical appointments for PLHIV. Many patients have missed HIV clinical visits, support meetings, follow-up tests, and counseling services [51–56]. In a multi-country survey, 55.8% of PLHIV were unable to meet their HIV physician face-to-face in the past month [51]. In Mexico, 44.3% of patients experienced follow-up failures due to structural barriers such as transportation difficulties and distance to the hospital [57]. In Peru, 37.2% reported difficulty accessing routine HIV care, with the most common reason being temporary closure of their primary HIV clinic [42]. A study in Atlanta (GA, USA) found that 19% of PLHIV had missed a scheduled HIV care appointment in the previous 30 days [58], while another study among men who have sex with men in 20 countries reported that 20% of PLHIV were unable to access their HIV care provider, even via telemedicine [27]. In contrast, this study documented a lower impact on non-compliance with medical consultations (11.9%), possibly due to the non-prolonged interruption of consultation services and greater regularization of services after the first year of the COVID-19 pandemic, as has been documented in other studies [41, 55]. Older age was significantly associated with missed visits, contrasting with COVID-19 pre-pandemic studies where missed visits were associated with younger age [59, 60]. Other COVID-19 pandemic-related factors such as inadequate transport, police abuse, insufficient transportation funds [61], lockdowns [62], limited access to health services, reduced income, inability to afford travel to health facilities or facemasks, fear of COVID-19 [54], and fear of visiting hospitals [55] could have had a greater impact on the life stability of older PLHIV. This instability has been correlated with a higher risk of missed medical appointments compared to their peers with less life chaos [63].

During the COVID-19 pandemic, ART-producing pharmaceutical companies faced challenges with international shipping due to border restrictions, transportation delays, increased lead times, and rising costs, contributing to global ART disruptions [64, 65]. However, surveys and observational studies have shown variability in ART refill interruptions. A global study in 20 countries reported that more than half were unable to access ART refills remotely, with the least access in Belarus, Brazil, Kazakhstan, Mexico, and Russia [27]. In Ethiopia, 27.4% of participants missed visits for refills [54], while in Peru, 24% reported difficulty picking up their ART due to cancelled appointments or lack of transportation [42]. A study in Italy documented a 23.1% decrease in dispensed ART during early 2020 compared to 2019, but this trend normalized after the first few months of the COVID-19 pandemic [41]. Similarly, a study in Haiti observed an 18% decline in ART refills [50], while in Brazil, only 17.2% reported an impact on ART refills [30]. In Taiwan, only 9.1% of PLHIV self-reported interrupted ART [48], while this study found that only 3% experienced interruption as a result of the COVID-19 pandemic, similar to reports from Brazil [43], Argentina [44], Northern Italy [41], and Indonesia [55] (4.2%, 3.9%, 3.2%, and 3%, respectively). A multi-country survey among PLHIV reported that 3.6% were unable to refill their ART [51], while a similar study in China found that 2.7% experienced interruption with a median duration of 3 (IQR 1–6) days and higher risk for those with a treatment abandonment history [66]. The low rate of interruption in this study may be due to continued operation of the ARV dispensary during the COVID-19 pandemic and the strategy of providing three months of ART at a time, as implemented in other countries [50, 66]. Thus, evidence of HIV care disruption and ART interruption during the COVID-19 pandemic was primarily during the early months and varied by region depending on measures implemented by each country. Despite these interruptions (self-reported or electronically recorded), adherence was maintained in several studies [30, 42, 43].

Although studies have shown discrepancies, PLHIV appear to be at high risk for adverse clinical outcomes from COVID-19, with some evidence of higher hospitalization and mortality rates [67–69]. Despite the effectiveness of COVID-19 vaccination in preventing these outcomes [14–17], almost half (44.5%) of participants in this study expressed vaccine hesitancy due to fear and mistrust, similar to reasons reported in Latin America [70], the USA, India, and China [71–73]. The rate of COVID-19 vaccine hesitancy among PLHIV in this study was lower than in Nigeria (57.7%) but higher than in India (38.4%) [73], France (28.7%) [74], China (27.5%) [75], Trinidad and Tobago (39%) [76], Brazil (23.9%) [51], and other Latin American countries (12.8%) [70]. Most patients with COVID-19 vaccine hesitancy were low-income young men with recent HIV diagnoses, consistent with other studies [19]. The highest proportion of COVID-19 vaccine hesitancy was found among those with recent diagnoses (< 1 year), possibly due to lack of knowledge about COVID-19 vaccination and HIV infection [77–79]. This highlights the importance of designing education strategies focused on COVID-19 vaccination in the context of HIV infection.

This study has several limitations. Firstly, the study is based on a non-probabilistic sample from a single center, which may accurately represent patients attending this specific center but may not reflect the broader population of PLHIV in Venezuela. This is despite the institution being the primary referral center for PLHIV in the country. Furthermore, the required and calculated sample size was not obtained, which restricts the statistical power to detect certain effects or differences. Secondly, the cross-sectional design limits causal inference and only provides a snapshot of challenges during a specific period of the COVID-19 pandemic. Additionally, information was collected at different times throughout the study period, so perceptions (e.g., vaccine willingness) may have been influenced by the rapidly evolving COVID-19 pandemic. Thirdly, the limited epidemiological surveillance on HIV in Venezuela over the past decade has posed significant challenges in acquiring data regarding the COVID-19 pre-pandemic period on newly diagnosed PLHIV, adherence, and undetectability rates, making a comparative analysis infeasible. Fourthly, while there was adequate weekly ART adherence in most patients, approximately one-third were unable to undergo viral load testing. This could be explained mainly by the limited availability of testing in the public healthcare sector and high cost in the private healthcare sector [5]. Fifthly, the limited access to CD4^+^ lymphocyte counting in the public system, coupled with the low income of PLHIV to afford private testing, resulted in a scarcity of T-CD4^+^ lymphocyte count results. This scarcity hindered our ability to correlate this value with other variables. Sixthly, some medical histories were incomplete or inadequate and were supplemented with direct patient questioning, introducing potential recall bias. Seventhly, while data on COVID-19 vaccine hesitancy is available, the specific reasons why they preferred not to be vaccinated were not thoroughly explored. The small sample size also limits the generalizability of these results. Finally, it was not possible to accurately calculate ART interruption and missed scheduled consultations from available records due to data quality issues.

## Conclusions

The disruption of HIV services during a public health crisis such as COVID-19 is an important problem for healthcare systems and policymakers to address, as it may exacerbate disparities in the HIV treatment cascade in settings with a high HIV burden or among vulnerable populations. This study found limited impact of the COVID-19 pandemic on adherence to consultations and interruptions of ART refills among PLHIV at the University Hospital of Caracas, Venezuela. However, due to the limitations of this study, it is essential to conduct comprehensive, multicenter studies with larger sample sizes that include regions with less access to the continuum of care for PLHIV.

## Data Availability

All data generated or analyzed during this study are included in the article.

## Abbreviations

HIV: Human immunodeficiency virus
PLHIV: People living with HIV
AIDS: Acquired immunodeficiency syndrome
ART: Antiretroviral therapy
WHO: World Health Organization
COVID-19: Coronavirus disease 2019
STDs: Sexually transmitted diseases
SD: Standard deviation
IQR: Interquartile range
TLD: Tenofovir disoproxil fumarate, lamivudine, and dolutegravir
NRTIs: Nucleoside reverse transcriptase inhibitors
NNRTIs: Non-nucleoside reverse transcriptase inhibitors
SARS-CoV-2: Severe acute respiratory syndrome coronavirus 2
